# Perceptions Regarding the Use of E-cigarettes Among Smokers in Saudi Arabia

**DOI:** 10.7759/cureus.70393

**Published:** 2024-09-28

**Authors:** Hani S Almugti, Noha A Alzahrani

**Affiliations:** 1 Primary Health Care, King Abdullah International Medical Research Center, King Saud bin Abdul-Aziz University for Health Sciences, Ministry of National Guard - Health Affairs, Jeddah, SAU; 2 Saudi Board of Preventive Medicine: Preventive Medicine, Ministry of National Guard - Health Affairs, Jeddah, SAU

**Keywords:** e-cigarettes, saudi arabia, smoker, smoking cessation, vaping

## Abstract

Background

Adults and young people select electronic cigarettes (ECs) due to their perceived lower risk compared to traditional combustible cigarettes.

Objective

This study aimed to measure the perceptions of the use of electronic cigarettes as a smoking cessation tool among the population of Saudi Arabia.

Methods

Between October 2023 and November 2023, we conducted a cross-sectional study of Saudi Arabia's general population over prominent social media platforms. We strictly adhered to the Strengthening the Reporting of Observational Studies in Epidemiology (STROBE) guidelines to report our results.

Results

Of the 376 total participants, the majority (89.6%) were male, with a median age of 35 years, and 96.3% were of Saudi nationality. Nearly half (47.3%) held Bachelor's degrees. Approximately 55.6% reported having previously attempted to quit smoking on multiple occasions. The predominant method for quitting was the use of ECs, which was reported by 88.7% of the participants. Nearly two-thirds (70%) perceived ECs as an effective cessation tool. Among those who had used EC to quit smoking, 62.5% had succeeded, while one-third (37.5%) were unsuccessful. The most frequently reported side effects were sore throat, changes in appetite, and headache, each of which affected ~50% of the participants.

Conclusion

This study highlights the complex relationship between EC use and smoking cessation among Saudi Arabian smokers. Although many users perceive ECs as effective cessation tools, their actual success rates in terms of long-term abstinence from smoking are questionable.

## Introduction

Tobacco addiction is a global public health problem that kills 8 million people annually [1]. Similarly, second-hand smoke kills 1.3 million people per year [1]. The early death of smokers reduces economic growth by reducing family income and increasing healthcare costs [1]. A significant number of individuals in Saudi Arabia (SA) have lost their lives because of tobacco consumption [2]. The prevalence of tobacco use in SA was approximately 20% in 2019, with electronic cigarette (EC) use accounting for approximately 3% [3]. The total cost of smoking and second-hand smoke in SA amounts to $17.2 billion, including both direct and indirect expenses [4].

In 2003, China developed ECs as a substitute for traditional cigarettes [5]. Multiple ECs are available on the market, but the fundamental elements of an EC include a battery, a compartment for e-liquids (a liquid combining flavoring, a solvent, and nicotine), a heating component, and a mouthpiece [6]. ECs have become popular among both adults and youth because they are perceived as being less toxic than combustible cigarettes [7,8]. Recent toxicological evidence indicates that ECs are not without risks but are considerably less damaging to the human body compared to traditional tobacco smoking [9]. This suggests a significant gap in evidence regarding the specific role of flavors in terms of reducing the public health burden of smoking [9].

The use of flavored EC liquids is of particular concern. Flavors that are attractive by name, description, advertising, and actual sensory experience may be particularly appealing to young people. This demographic is generally receptive to compelling descriptions and flavor names [10] and may prefer sweet flavors because of their more sensitive taste buds [11]. There is concern in the literature and among policymakers that flavored e-liquids may promote the use of ECs among young nicotine-free consumers, which could then serve as an entry point to smoking [12]. A popular counterpoint to this argument claims that attractively flavored ECs may displace traditional tobacco smoking because they provide an alternative [13].

Despite the growing body of literature on EC use, few studies in the literature specifically focus on EC use in the Saudi Arabian population. Understanding the perceptions and effectiveness of ECs as smoking cessation tools in this context is crucial, given the unique cultural and social factors that may influence smoking behaviors and cessation outcomes. This study aimed to address this gap by evaluating the general population's perception of EC use as a smoking cessation tool in Saudi Arabia.

## Materials and methods

Study design and setting

This cross-sectional study was conducted between October 2023 and November 2023. An electronic questionnaire created via Google Forms (Google LLC, California, USA) was distributed through the most popular social media platforms in Saudi Arabia, i.e., WhatsApp, Telegram, and Twitter (X), for contacting participants. 

Ethical considerations

The institutional review board of King Abdullah International Medical Research Center (KAIMRC) in Jeddah, Saudi Arabia, approved this study with IRB number (2167/23). To ensure comprehensive and accurate reporting, we strictly adhered to the Strengthening the Reporting of Observational Studies in Epidemiology (STROBE) guidelines when reporting our results.

Subjects

Our survey targeted smokers of all ages and sexes in Saudi Arabia who had transitioned to using ECs (or “vaping”) as smoking cessation aids.

A subset of participants were excluded from the analysis. These included those who did not complete the questionnaire, as well as those who had not switched from traditional cigarettes, hookahs, or smokeless tobacco to ECs.

Sample size and sampling technique

The sample size was calculated using the Raosoft sample size calculator (Raosoft Inc., Seattle, Washington, USA). A 26% prevalence of EC use, 95% confidence interval (CI), and 5% margin of error were calculated. The initial sample size of 292 participants was increased to 350 to account for missing data or non-respondents. The questionnaire was sent to EC users using a nonprobability sampling method (snowball sampling).

Data collection methods, instruments used, and measurements

At the end of the survey period, 659 responses were obtained. Of these, 277 were from non-smokers and smokers who had not switched to ECs. This left 382 responses from smokers who switched to ECs that met our inclusion criteria. We analyzed responses from 376 participants, after excluding 6 because they had not completed the questionnaire.

Questionnaire

To formulate the questionnaire for this study, we conducted a comprehensive review of similar relevant questionnaires in the literature, as well as interviews with smokers who had transitioned to vaping. We aimed to gather information regarding their experiences during their initial trials of vaping, as well as after they switched from traditional cigarette smoking to vaping. Physicians specializing in preventive medicine and public health reviewed the questionnaire to ensure its accuracy and validity. To verify the clarity of the questionnaire and assess its completion time, we conducted a preliminary study involving 10 smokers.

The questionnaire for this study was an online, self-administered survey comprising five sections. The first contained two exclusion questions. The second section collected sociodemographic and occupational information. The third section introduced smoking cessation and quitting methods. The fourth section comprised an insight assessment to evaluate EC use. The fifth section contained information regarding the side effects experienced by EC users, the improvements in such side effects compared to regular cigarette use, and a source for further information regarding these side effects.

Data management and analysis plan 

The data were entered and analyzed using SPSS statistical software version 21.0 (IBM Corp., Armonk, NY, USA). The combination of data entry and coding resulted in improved data quality. Categorical variables are displayed as frequencies and percentages, whereas age is represented by medians and interquartile ranges (IQRs). Statistical significance was evaluated using the chi-squared, Fisher’s exact, and Mann-Whitney U tests. Logistic regression analysis was applied to evaluate the major factors that influenced the respondents’ perceptions of ECs as effective tools for quitting smoking.

## Results

Demographic information

Table 1 presents the main characteristics of the study population. Between October and November of 2023, we received responses from 376 participants who completed the questionnaire and met our inclusion criteria. The median age of the participants was 35 years. Of the 376 total participants, 89.6% were male and 96.3% were of Saudi nationality. The majority (47.3%) had Bachelor's degrees, followed by diplomas (22.3%), high school or less (17.1%), and other forms of higher education (13.3%). In terms of occupational statuses, most of the respondents were employees (64.4%) and students (16%) while the remainder were not employed or were retired.

**Table 1:** Demographic characteristics of study participants

Variable		Median	Range
Age		35	16–79
		Frequency	Percentage
Sex	Male	304	89.6
	Female	72	18.8
Nationality	Saudi	368	96.3
	Non-Saudi	8	2.1
Education level	High school or less	64	17.1
	Diploma	84	22.3
	Bachelor's degree	178	47.3
	Higher education	50	13.3
Job	Student	60	16
	Employee	242	64.4
	Unemployed	33	8.8
	Retired	41	10.9

Table 2 illustrates the history of tobacco cessation among EC users. More than half (55.6%) of the participants reported that they had quit smoking at least once. By contrast, a smaller proportion (40.7%) reported quitting only once, whereas 3.7% reported that they had never quit. Notably, the most common method reported for quitting was ECs (88.7%), whereas similar proportions were reported for nicotine patches and prescribed medications, e.g., varenicline or bupropion.

**Table 2:** Smoking history of participants

Smoking history	Answer	Frequency	Percentage
Previous quitting attempts (N = 376)	Never	14	3.7
	One Time	153	40.7
	More Than One Time	209	55.6
What quitting methods did you use? (N = 362)			
Gum or lozenges	No	291	80.4
	Yes	71	19.6
Nicotine patch	No	250	69.1
	Yes	112	30.9
Medication (e.g., varenicline or bupropion)	No	255	70.4
	Yes	107	29.6
E-cigarettes (ECs, vape)	No	41	11.3
	Yes	321	88.7
Stop immediately without any aid	No	301	83.1
	Yes	61	16.9

**Figure 1:**
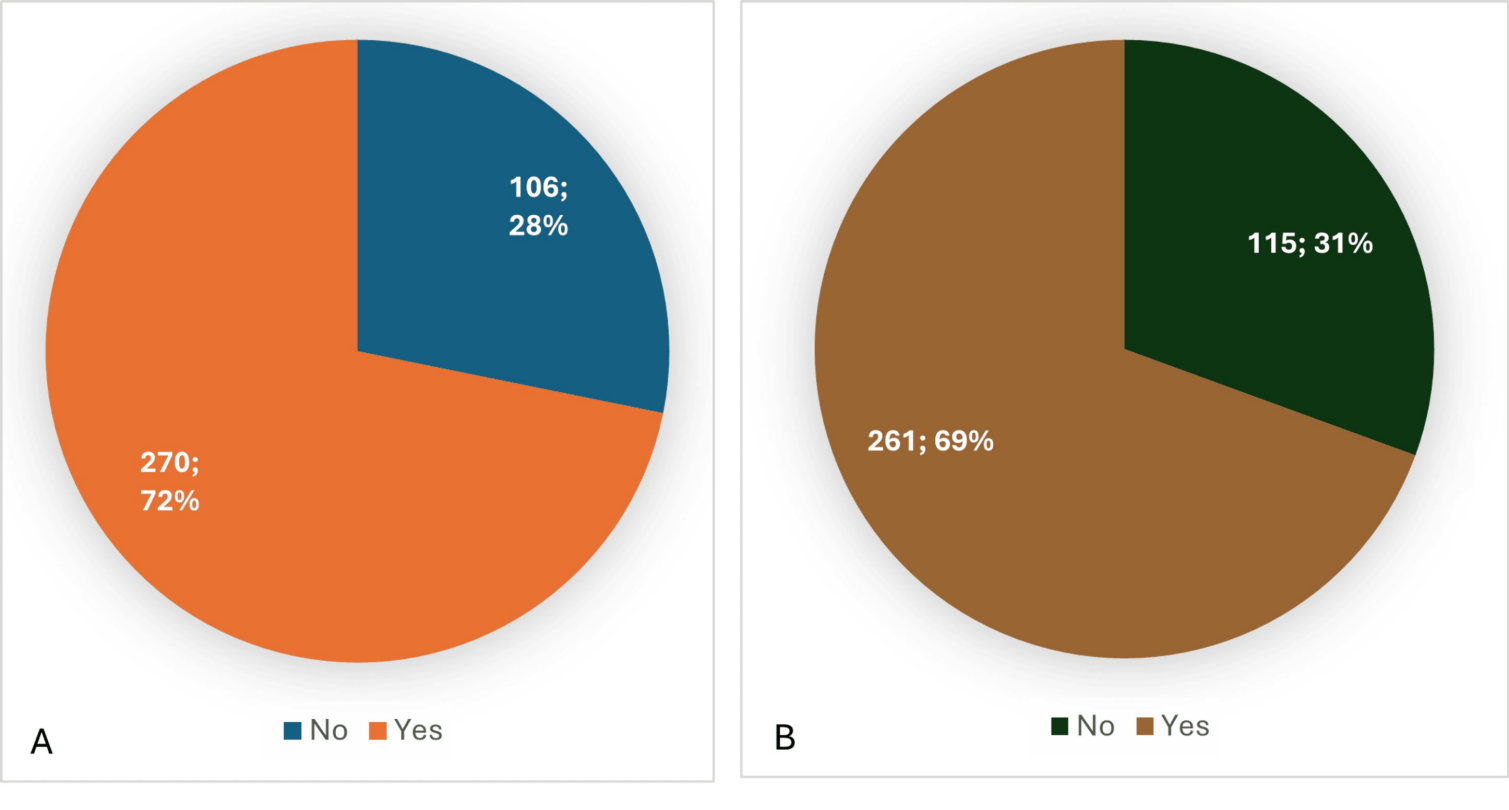
A: Do you think people who use E-cigarettes are considered smokers?; B: Do you think E-cigarettes are a tool to quit smoking? (number; percentage)

Figure 1A shows that 72% of the participants thought that individuals who used ECs should be classified as smokers. Moreover, Figure 1B illustrates that nearly 70% of the people in our sample perceived ECs as an effective way to quit smoking.

EC use

**Figure 2:**
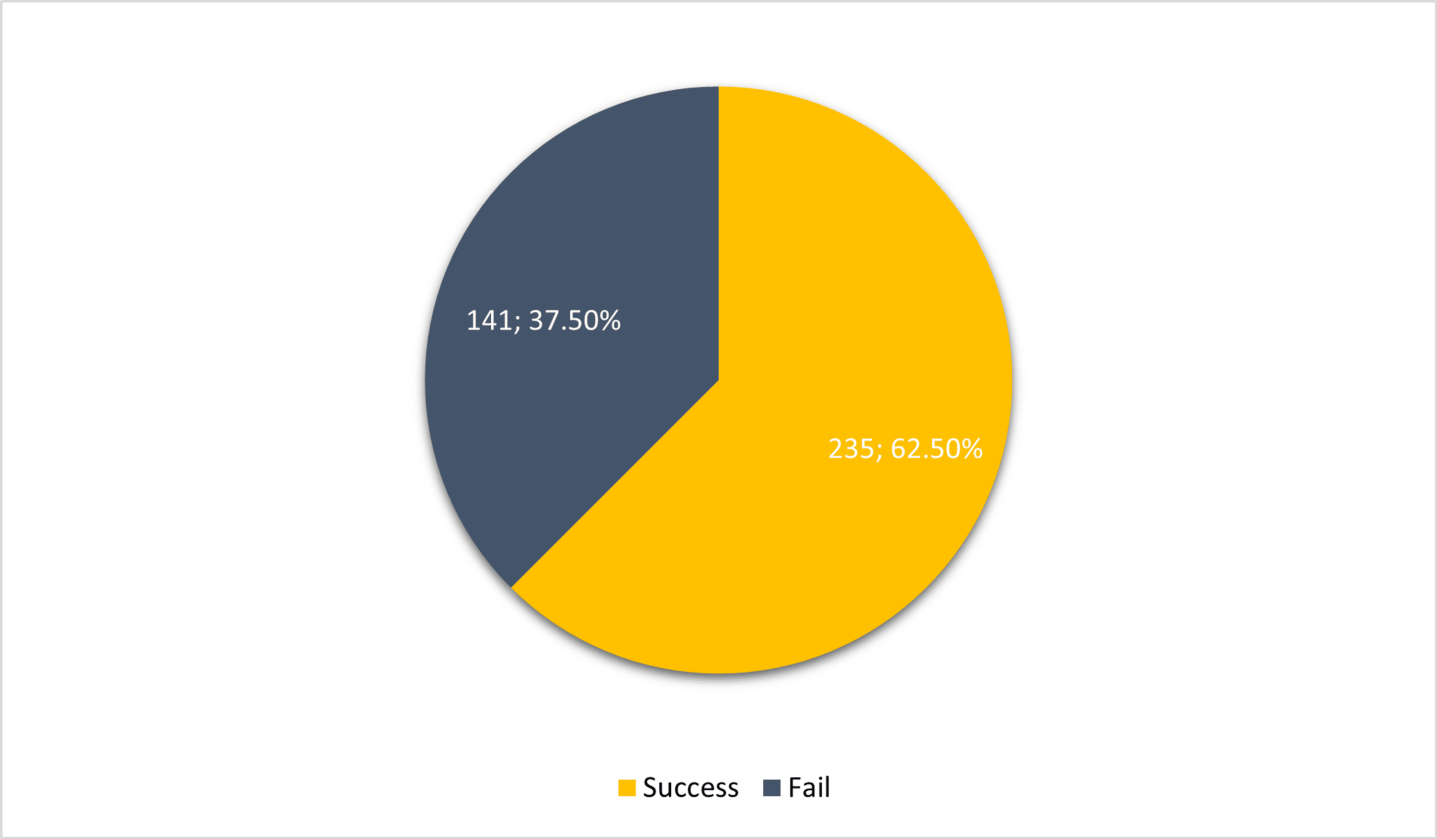
The percentage of participants who successfully quit smoking using E-cigarettes as compared to the failure rate (number; percentage)

Figure 2 indicates that individuals who used ECs to quit smoking achieved a success rate of 62.5%, whereas 37.5% were unsuccessful in their attempts to quit.

**Figure 3:**
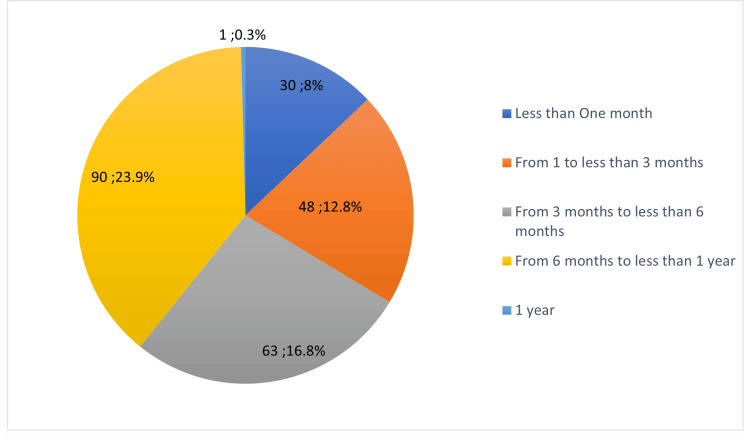
Perceptions regarding the amount of time smokers use E-cigarettes before fully quitting smoking (number; percentage)

It is apparent from Figure 3 that approximately 25% of the respondents used ECs for 6-12 months before they were able to quit smoking. However, approximately 40% of those who used ECs to help them stop smoking were actively using ECs at the time of the survey and had therefore not been able to successfully quit smoking.

**Figure 4:**
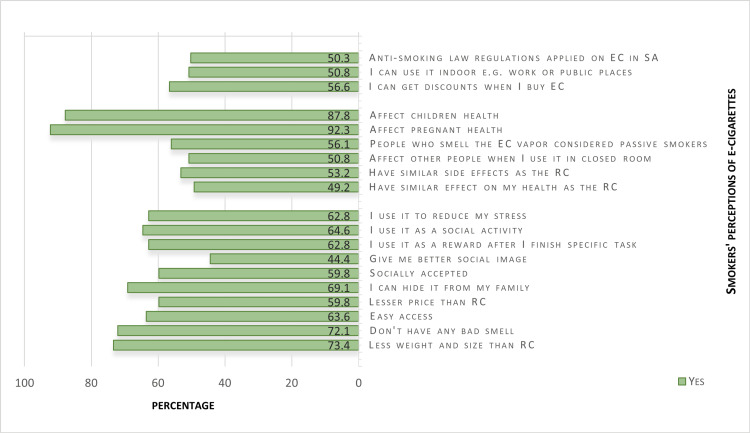
Smokers' perceptions of ECs * ECs = E-cigarettes; RCs = regular cigarettes; Task = work or study; SA = Saudi Arabia

Regarding their perceptions of EC use, the majority of the EC users (60%) believed that ECs were less expensive than traditional cigarettes, and 73.4% perceived them as being lighter. Approximately 65% used ECs for socialization purposes while 62.8% used them to relieve stress or as a form of reward for hard work. Additionally, 69% reported that they were able to keep their EC use hidden from their families and nearly 60% engaged in it because it was socially acceptable. In terms of the health effects of ECs, 92% of the respondents thought that they negatively impacted pregnant women while 87% believed that they negatively affected children. Regarding the regulation of ECs, half of the respondents believed that they had permission to use them in public indoor areas and that ECs were subject to the same restrictions as conventional cigarettes in terms of anti-smoking legislation (Figure 4).

**Figure 5:**
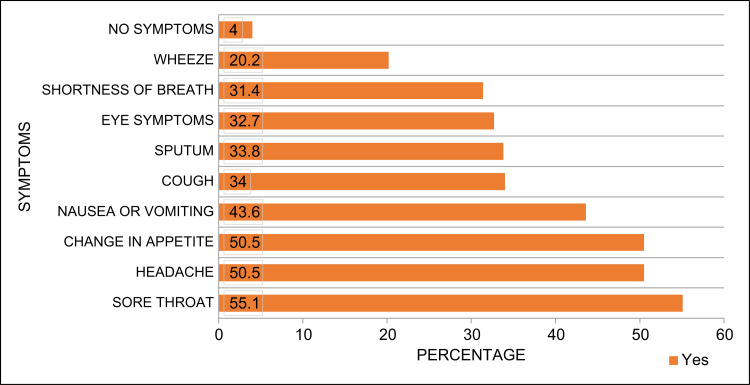
Side effects of e-cigarette use

Figure 5 shows a summary of the prevalent symptoms reported as side effects following the use of ECs. These included sore throat (55%), change in appetite (50.5%), and headache (50.5%).

The most notable finding in Table 3 is that 71.5% of the respondents indicated that they obtained their information from personal experiences while using ECs, while 36.2% cited friends and social media as information sources.

**Table 3:** Sources of information related to e-cigarettes

Source of information	Frequency	Percentage
Friends	136	36.2
Personal experience	269	71.5
Social media	136	36.2
Physician	59	15.7
Scientific reference	79	21.0

Relationships between EC perceptions, demographic characteristics, and other factors

Table 4 details the association between demographic data, previous quitting attempts, different methods used to quit smoking, and the perceptions of smokers regarding the effectiveness of ECs as a smoking cessation aid, as well as the classification of ECs users as smokers. We found statistically significant differences in terms of sex (p = 0.016), educational level (p = 0.017), previous quitting attempts (p < 0.01), use of gum or lozenges (p = 0.04), use of ECs (p = 0.008), immediate cessation of smoking (p = 0.026), and the views of ECs users as smokers. However, only educational level (p < 0.01), prior attempts to quit smoking (p = 0.002), use of ECs (p = 0.01), and immediate cessation (p = 0.003) were significantly associated with the perception of ECs as a smoking cessation tool.

**Table 4:** Association among demographic factors, smoking cessation methods, and individual perceptions of E-cigarette consumption 'Mann-Whitney U test, *significant p-value, ^Fisher’s exact test

Variable	Perceptions of e-cigarette users as smokers		Perceptions of e-cigarette use as a smoking cessation tool	
	X^2^	p-value	X^2^	p-value
Age	13,159.5^'^	0.225	14,684^'^	0.739
Sex	5.843	0.016^*^	0.000	0.995
Nationality		0.450^^^		1.0^^^
Education level	12.077	0.017^*^^	20.862	< 0.01^*^^
Job	1.123	0.771	0.642	0.887
Previous quitting attempts		< 0.01^*^^		0.002^*^^
What quitting methods did you use? (N = 362)				
Gum or lozenges	4.222	0.04^*^	0.427	0.514
Nicotine patch	0.737	0.391	0.305	0.581
Medication (e.g. varenicline or bupropion)	1.979	0.16	0.995	0.319
E-cigarette (vape)	6.971	0.008^*^	19.072	< 0.01^*^
Stop immediately without any aid	4.929	0.026^*^	8.972	0.003^*^

A logistic regression model (Table 5) was used to predict insights regarding EC use as a method to quit smoking. This was done using the backward Wald method with factors including age, sex, educational level, occupation, and attempts to quit smoking.

**Table 5:** Binary logistic regression analysis of the predictor variables related to insight into e-cigarettes as a way to quit smoking

Characteristics	P-value	Odds Ratio (OR)	95% Confidence Interval (CI)	
Education level (Diploma and higher)	0.002	0.407	0.228	0.725
Previous quitting attempts (More Than One Time)	0.001	2.329	1.428	3.8
Constant	<0.01	1.965		

When controlling for other predictor variables, smokers who reported having attempted to quit smoking more than once were twice as likely to consider vaping as a smoking cessation aid (odds ratio (OR) = 2.4, 95% CI = 1.47-3.96, p < 0.05). Those who had diploma degrees or higher educational levels were 61% less likely to perceive ECs use as a smoking cessation tool than those with high school degrees or less (OR = 0.397, 95% CI = 0.22-0.716, p = 0.002).

## Discussion

This study assessed the perceptions and experiences of EC use among smokers in Saudi Arabia. The findings revealed significant insights into demographic factors, smoking cessation attempts, and the overall perception of ECs as tools for quitting smoking.

Our demographic analysis indicated that most of the ECs users surveyed were male with a median age of 35 years. This aligns with previous studies, such as those by Alzahrani et al., who also found a higher prevalence of male EC users in Saudi Arabia [14,15]. This male predominance may be attributable to cultural norms and societal smoking-related behaviors in the region [15,16]. Our analysis of educational backgrounds showed that most of the users had Bachelor’s degrees, suggesting that higher educational levels may be associated with EC use, potentially owing to increased awareness and access to information regarding smoking alternatives [14,15].

A significant proportion of the participants (55.6%) reported having made multiple attempts to quit smoking, with ECs being the most commonly used method (88.7%). This finding is consistent with global trends, which have shown that ECs are often used as smoking cessation tools [17]. However, this study also revealed that despite using ECs, a considerable number of users continued to smoke or returned to smoking traditional cigarettes, highlighting the mixed effectiveness of ECs for achieving long-term smoking cessation [18].

The study showed that nearly 70% of the participants viewed ECs as an effective method for quitting smoking; however, only 62.5% reported successfully quitting smoking using ECs. This discrepancy suggests that although ECs are perceived positively, their actual effectiveness in aiding smoking cessation varies. The reasons for this variation may include factors such as nicotine addiction, psychological dependence, and social environment, as indicated by the high percentage of our respondents (60%) who believed that ECs were less expensive and more socially acceptable than traditional cigarettes [15].

Most of the participants believed that ECs posed significant health risks, particularly to pregnant women and children. This aligns with findings from other studies that have highlighted the potential health hazards associated with EC use such as respiratory issues and nicotine addiction [19]. The most commonly reported side effects of EC use in this study were sore throat, changes in appetite, and headaches, which are consistent with the symptoms reported in other studies [20]. These health concerns underscore the need for more comprehensive public health education regarding the risks of EC use [21].

Logistic regression analysis revealed that educational level and previous attempts to quit smoking were significant predictors of perceptions regarding ECs. Individuals with higher educational levels were less likely to view ECs as effective cessation tools, possibly because of their greater levels of awareness regarding the associated health risks. Conversely, those who had attempted to quit smoking multiple times were more likely to consider ECs as helpful, reflecting their ongoing search for effective cessation methods.

Limitations

This study was subject to several key limitations worth noting. First, the use of a cross-sectional design limited our ability to establish causality between EC use and smoking cessation. Second, the reliance on self-reported data may have introduced some bias, as the participants may have under-reported their smoking behaviors or over-reported their cessation success. Third, the study sample, which was primarily recruited through social media, may not be fully representative of the broader population of smokers in Saudi Arabia. Additionally, the sample size was relatively small, which may limit the generalizability of our findings to other populations. Finally, the study did not account for the long-term health outcomes of EC use, which warrant further longitudinal examination.

## Conclusions

This study highlights the complex relationship between EC use and smoking cessation among smokers in Saudi Arabia. Although many users perceive ECs as effective cessation tools, their actual success in terms of promoting long-term smoking abstinence remains questionable. This study highlights the importance of targeted public health interventions and policies to educate smokers regarding the potential risks and benefits of EC use, particularly focusing on high-risk groups such as young adults and individuals with lower educational backgrounds.
